# Phosphorylated fibronectin enhances cell attachment and upregulates mechanical cell functions

**DOI:** 10.1371/journal.pone.0218893

**Published:** 2019-07-10

**Authors:** Garif Yalak, Jau-Ye Shiu, Ingmar Schoen, Maria Mitsi, Viola Vogel

**Affiliations:** Laboratory of Applied Mechanobiology, Institute of Translational Medicine, Department of Health Sciences and Technology, ETH Zürich, Zurich, Switzerland; Seoul National University College of Pharmacy, REPUBLIC OF KOREA

## Abstract

A large number of extracellular matrix proteins have been found in phosphorylated states, yet little is known about how the phosphorylation of extracellular matrix proteins might affect cell functions. We thus tested the hypothesis whether the phosphorylation of fibronectin, a major adhesion protein, affects cell behavior. Controlled *in vitro* phosphorylation of fibronectin by a casein kinase II (CKII) significantly upregulated cell traction forces and total strain energy generated by fibroblasts on nanopillar arrays, and consequently other elementary cell functions including cell spreading and metabolic activity. Mass spectrometry of plasma fibronectin from healthy human donors then identified a constitutively phosphorylated site in the C-terminus, and numerous other residues that became phosphorylated by the CKII kinase in vitro. Our findings open up novel strategies for translational applications including targeting diseased ECM, or to develop assays that probe the phosphorylation state of the ECM or blood as potential cancer markers.

## Introduction

While the phosphorylation of intracellular proteins regulates intracellular signaling, comparatively little is known whether extracellular matrix (ECM) proteins exist in phosphorylated states and whether this is of physiological relevance. A comprehensive previous analysis of existing mass spectrometry (MS) data revealed that a large number of ECM proteins, including but not limited to vitronectin, laminin-1, osteopontin, bone sialoprotein, collagens and fibronectin can exist in phosphorylated states [1–6]. Since fibronectin is a major component of the ECM, which is upregulated during development, wound healing and cancer [7], we previously mined proteomic data and identified around 50 residues on fibronectin, that were reported to be phosphorylated for fibronectin isolated from human blood and various cancer tissue types [2]. Fibronectin has been previously described to be phosphorylated by a casein kinase II (CKII)-like protein kinase [8], however, the effect of the phosphorylation of extracellular proteins on potential physiological cell functions remains elusive. Thus, we asked the principle question whether the phosphorylation of fibronectin could alter basic mechanical cell functions. We asked this question in the context of fibroblasts, which are the main producers and remodelers of ECM. Fibroblasts assemble both forms of fibronectin, i.e. plasma fibronectin that circulates in the blood, and cellular fibronectin which they produce on their own (for reviews see [7, 9]), into fibrillar ECM in connective tissues, wound sites and cancer stroma. In search for potential physiological effects of phosphorylated fibronectin, we have quantified alterations in cell functions on surfaces that were either coated with native, phosphorylated or dephosphorylated human plasma fibronectin under otherwise identical cell culture conditions. To modulate the phosphorylation of human plasma fibronectin, we have chosen CKII to induce fibronectin phosphorylation, as fibronectin has been previously reported to be phosphorylated by a CKII-like protein kinase [8], and alkaline phosphatase (AP) to dephosphorylate fibronectin. In the following, we studied cell spreading, proliferation, migration and metabolic activity, as well as traction forces on these fibronectin surface coatings, characterized the location of phosphorylation sites on fibronectin, and are seeking for possible explanations of the observed behavioral changes.

## Results

### Cell spreading and metabolic activity are enhanced on CKII-phosphorylated fibronectin

**Fig 1:**
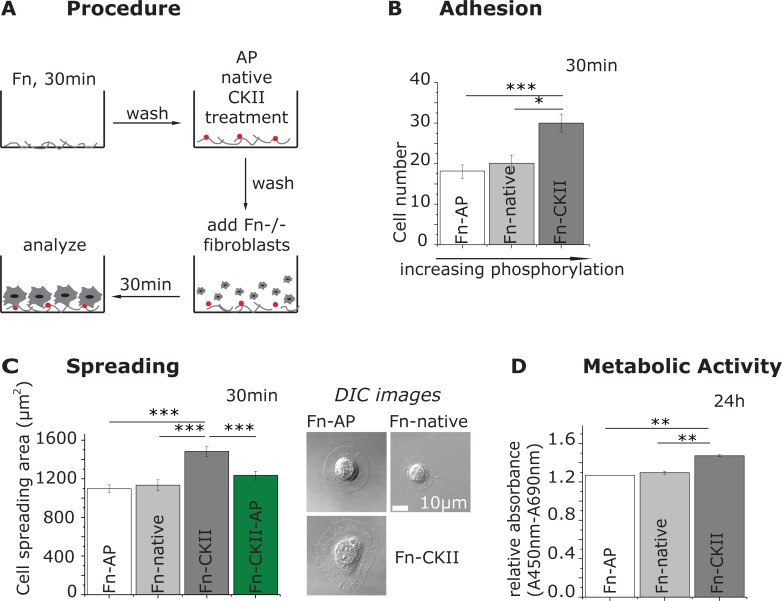
Effect of fibronectin phosphorylation on cell adhesion, spreading and metabolic activity on flat substrates. **A:** Schematic of the experimental procedure. Fibronectin-coated surfaces were treated by either alkaline phosphatase (Fn-AP), kept native (Fn-native), or by casein kinase II (Fn-CKII). Fn(-/-) mouse fibroblasts were seeded and analyzed. **B:** Number of adherent cells and **C** cell spreading area and representative DIC micrographs taken 30 min after cell seeding. Depicted are the mean and standard deviation of 200 cells from duplicates in two independent experiments. **D:** Metabolic activity of the fibroblasts 24 h after seeding using the standard cell proliferation reagent WST-1. Statistical comparison done with Student’s *t*-test between pair of samples connected with lines on the column plots: ***, p-value<0.005; **, p-value<0.01; *, p-value<0.05; not significant (ns).

To ask the principle question whether the phosphorylation of human plasma fibronectin can indeed alter basic mechanical cell functions in cell culture, human fibronectin was first adsorbed to glass bottom dishes and subsequently either phosphorylated *in vitro* by CKII (Fn-CKII), or dephosphorylated by alkaline phosphatase (AP; Fn-AP), or left as-is (Fn-native) (Fig 1A). This sequence of sample preparation was chosen to ensure that all surfaces, treated and untreated, had the same amount of surface adsorbed fibronectin. To further exclude effects from endogenous cellular fibronectin of unknown phosphorylation state, we used mouse fibronectin knock-out (Fn^-/-^) fibroblasts throughout the study as they are not able to express fibronectin on their own [10]. Finally, these fibroblasts were seeded on these substrates, cultured for the specified durations, washed, fixed and analyzed.

To assess various cell functions, we first analyzed the number of adherent cells 30 min after seeding, washing and fixing the cells. Compared to the untreated fibronectin substrates, CKII phosphorylation significantly increased the number (average cell number: 30) of adherent fibroblasts by ~60% for the same seeding densities, whereas dephosphorylation (average cell number: 18) had no pronounced effect compared to the native fibronectin control (average cell number: 20) (Fig 1B). Simultaneously, the cell spreading area was significantly increased by ~35% on the Fn-CKII substrates (average area: 1480 μm^2^), but unaltered on the Fn-AP samples (average area: 1100 μm^2^) (Fig 1C, Figure A in S1 File). In a control, phosphorylated Fn-CKII was subsequently dephosphorylated with alkaline phosphatase (Fn-CKII-AP) which significantly reduced the spreading area by roughly 20% compared to the Fn-CKII samples down to a level that was comparable to the Fn-AP surfaces (Fig 1C).

To ask whether the increased cell spreading is accompanied by a change of cell metabolic activity, we cultured the fibroblasts for 24 h and then quantified their metabolic activity by the WST-1 cell proliferation assay (see Methods). A significant enhancement of metabolic activity on CKII-phosphorylated fibronectin (Fn-CKII) was observed, whereas no difference could be detected between native fibronectin (Fn-native) and dephosphorylated fibronectin (Fn-AP) samples (Fig 1D). In contrast, we did not find a major effect of fibronectin phosphorylation on cell proliferation and migration (Figure B in S1 File). In summary, our data show that CKII-phosphorylated fibronectin enhances fibroblast spreading and metabolic activity.

### Phosphorylated fibronectin enhances cell traction forces

**Fig 2:**
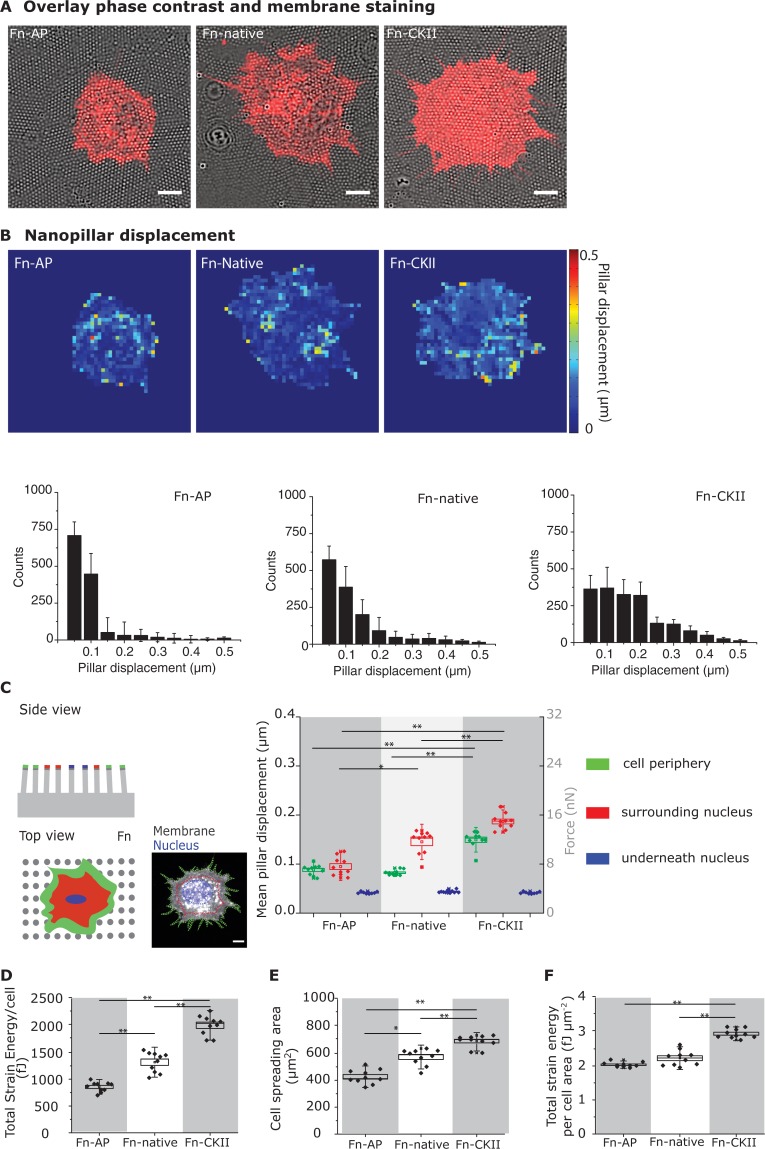
Effect of fibronectin phosphorylation on cell traction forces as probed by Fn-coated nanopillar arrays. Fn(-/-) mouse fibroblasts were cultured for 30 min after seeding on fibronectin-coated nanopillar arrays as described above (Fig 1A). These nanopillar arrays were fabricated from SU-8 and the pillars had a spring constant of 79 nN/μm. **A**: Overlay of DIC micrographs (gray) and fluorescence images with a DiI membrane stain (red). Scale bars: 10 μm. **B:** Heat maps of pillar displacements and corresponding histograms as caused by single cells. **C:** Sub-cellular distribution of traction forces. Depicted are mean pillar displacements (left axis) and corresponding traction forces (right axis) for the cell periphery (green; area 5 μm from edge), the region underneath the nucleus (blue), and the region in-between (red). **D:** Total strain energy per cell. **E:** Cell spreading area on the nanopillar arrays. **F:** Strain energy per spreading area. For **B-F**, n = 10 cells were analyzed per condition. Statistical comparison done with Student’s *t*-test between pair of samples connected with lines on the column plots: ***, p-value<0.005; **, p-value<0.01; *, p-value<0.05; not significant (ns).

Since cell morphology correlates with cell contractility, and since cell adhesions mature more rapidly if held under tension[11], we next asked whether cell traction forces and respective total strain energies of single cells are affected by the phosphorylation of surface-bound fibronectin. To map cell traction forces with high spatial resolution, we exploited recently developed nanopillar arrays [12, 13]. The nanopillar tip surfaces were thereby first coated with native plasma fibronectin that was subsequently treated with AP or CKII using the above-mentioned protocol. Fibroblasts were allowed to spread on the nanopillar arrays for 30 min after seeding. They spread on top of the nanopillars and showed increased spreading area as well as enhanced pillar displacements on phosphorylated fibronectin, while the dephosphorylation of native fibronectin caused a reduction of the cell spreading area and pillar displacements (Fn-AP < Fn-native < Fn-CKII; Fig 2A and 2B). Recently, we found for fibroblasts on nanopillar arrays that the largest displacements of nanopillars coated with native plasma fibronectin are found not at the cell periphery, but in the perinuclear regions where highly tensed actin stress fibers that cross the nucleus are anchored and pull on the nanopillars [13]. Since these actin fibers have typical diameters of more than a micrometer, each of them contacts several nanopillars at once, and since those forces are acting at a steep angle, a stress fiber anchored to several nanopillars pulls them into pillar clusters [13]. Thus, we next analyzed the nanopillar displacements within a 5-μm-wide rim at the cell periphery, which we know from our previous study to be rich in β3-integrins, while the perinuclear region is gradually enriched in α5β1-integrins [13]. As observed before for native fibronectin, the pillar displacements are highest in the perinuclear region, while they are close to thermal noise underneath the cell nucleus [13]. Most importantly, CKII phosphorylation of fibronectin upregulates both the peripheral and the perinuclear pillar displacements (Fig 2C), clearly showing that the phosphorylation of fibronectin upregulates cell traction forces. Surprisingly dephosphorylation of native fibronectin by AP reduced the perinuclear pillar displacements to the same levels as seen at the cell periphery.

The absolute cell spreading areas on nanopillars for the different conditions, Fn-AP, Fn-native and Fn-CKII, were significantly smaller than those on flat substrates (Fig 1C), as observed before [12, 13]. Our data further show that enhanced cell spreading correlates with enhanced total strain energy per cell (Fig 2D and 2E), as well as with the total strain energy normalized per unit area (Fig 2F). Phosphorylation of nanopillar-bound fibronectin by CKII increased the total strain energy per cell by 55% compared to native fibronectin, whereas dephosphorylation of native fibronectin decreased the strain energy by 32% (Fig 2D). However, normalizing the strain energy to unit cell area revealed similar values on Fn-native and Fn-AP, but significantly elevated (+35%) ones on CKII treated Fn (Fig 2F).

### Plasma fibronectin from healthy donors contains a phosphorylated residue S2384 recognized by the Golgi serine/threonine protein kinase Fam20C

**Fig 3:**
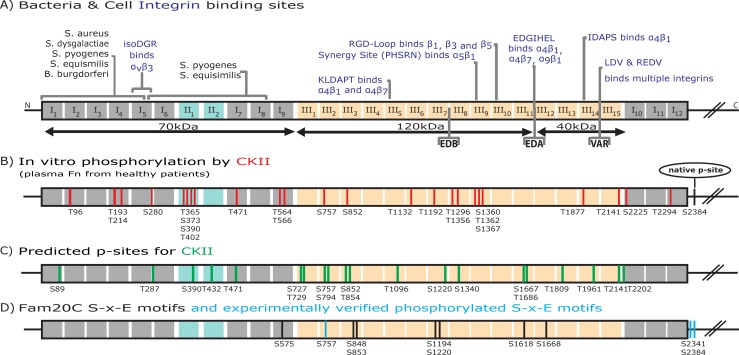
Predicted and experimentally verified phosphorylation sites on fibronectin. **A:** Schematic representation of plasma fibronectin isoform 1 with modules type I (gray), type II (turquoise) and type III (orange). Proteolytic fragments and cell binding sites are indicated. (See Section A in S1 File). The proteolytic fragments are derived from plasma fibronectin and thus do not contain the alternatively spliced domains- **B:** Fibronectin phosphorylation sites as identified by mass spectrometry (MS) after phosphorylating human plasma fibronectin in solution by CKII (red). **C:** Phosphorylation sites as predicted for CKII using the server NetPhosK. **D:** Analysis of the positions of S-x-E motifs as potential recognition sites for the Fam20C kinase and experimentally verified phosphorylated residues located in a S-x-E motif (blue).

Fibronectin contains a large number of binding sites for bacteria and cells, as well as for growth factors and other ECM constituents (Fig 3A, Figure C in S1 File). Phosphorylation on or close to these binding sites may have a regulatory effect on these interactions. With the aim to map the existence of phosphorylated sites on native plasma fibronectin isolated from human blood of healthy donors, a mass spectrometry analysis was performed on pooled blood received from the Blood Donation Center Zurich. Within the five human samples that we obtained from the Blood Donation Center Zurich, we consistently found that native fibronectin was phosphorylated at residue S2384, located near the C-terminal region of fibronectin, close to the inter-chain disulfides bond (Fig 3B). In fact, the phosphorylated site S2384, as found in our study, has also been identified previously in the literature where it was shown that S2384 is phosphorylated by the Golgi serine/threonine protein kinase Fam20C in different cell lines [14]. Both kinases belong to the same family of kinases. Nothing is known so far, whether this phosphorylation plays any physiological role.

### CKII phosphorylates fibronectin *in vitro* at multiple sites

Mass spectrometry was then used to identify residues of fibronectin that are phosphorylated *in vitro* when fibronectin or fibronectin fragments in solution were exposed to the kinase CKII. When we treated dimeric fibronectin in solution with CKII, we found that only the S2384 site was phosphorylated (Table A in S1 File), thus, only the same site that we also found to be phosphorylated in human blood from healthy donors. When we enzymatically cleaved fibronectin prior to the CKII-treatment, we found 24 different phosphorylated sites in CKII-treated fibronectin (Fig 3B, Table A in S1 File), in addition to the site S2384. Many of the CKII phosphorylated residues are clustered in module FnIII_9_ harboring the synergy site, namely S1360/T1362/S1367. The synergy site distinguishes fibronectin from other adhesion proteins and strengthens the binding of α5β1 integrins to fibronectin. Other phospho-site clusters exist on the N-terminal FnII_1_ module and several phospho-sites are located in FnI modules that are essential for fibronectin fibrillogenesis and also contain various bacterial binding sites [15]. We thus conclude that many of fibronectin’s phosphorylation sites are buried in the compact structure in which dimeric fibronectin circulates in the blood stream. Many cryptic binding sites can become exposed upon protein fragmentation or upon other conformational changes that expose otherwise cryptic binding sites [9]. It is also well known that fibronectin undergoes major conformational changes towards more extended states upon adsorption to surfaces or during fibrillogenesis, both of which open up many cryptic sites that are buried in its soluble compact configuration.

Since the sequence motif phosphorylated by CKII is known, we next utilized two bioinformatics prediction servers (NetPhosK 1.0 and NetPhos 2.0) and found a number of sites that are predicted to have the potency to be phosphorylated by the protein kinase CKII, as well as by other protein kinases (Fig 3C; Table B in S1 File). NetPhosK 1.0 correctly predicted five of our experimentally detected phospho-sites by CKII, specifically as CKII sites, whereas it predicted the other remaining 19 experimental phospho-sites by CKII without specifying the kinase. The fact that not all predicted phosphorylation sites were experimentally detected could be due to the limited coverage and sensitivity of the MS analysis, the limited kinase activity in our assay, the inaccessibility of the predicted sites as they might get buried in the structure of the protein as discussed above or by the accuracy and limits of the prediction software.

Finally, the entire fibronectin sequence contains a total of 10 S-x-E motifs that are recognized by the secreted Fam20C kinase, of which only 3 correspond to the phosphorylated sites found in this study and in data published earlier [2] (Fig 3D). We conclude that the native fibronectin isolated from blood of healthy donors is already phosphorylated on S2384 and can be phosphorylated at additional multiple locations by CKII.

### Phosphorylation sites located in the 120 kDa fragment (FnIII_1-11_), containing the synergy-RGD binding site, contribute to the enhanced fibroblast spreading on phosphorylated fibronectin

To narrow down the specific locations of phosphorylated residues that contribute to the observed alterations in cell functions, we coated the surfaces with the following commercially available proteolytic fibronectin fragments (as defined in Fig 3A): the N-terminal 70 kDa fragment (FnI_1-9_), containing an iso-DGR integrin binding site for αvβ3 on module FnI_5_, the 120 kDa fragment (FnIII_1-11_), which contains the synergy and RGD-binding sites on FnIII_9_ and FnIII_10_ respectively, and the C-terminal 40 kDa (FnIII_12-14_), which contains the growth factor and the major heparin binding site.

**Fig 4:**
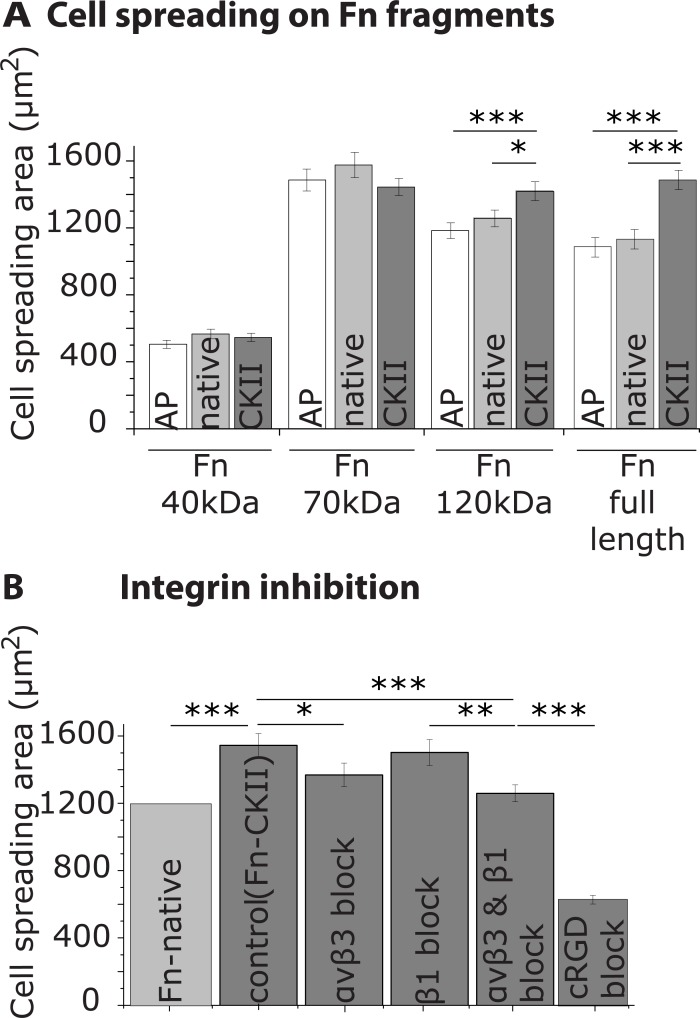
Relative contributions of fibronectin fragments and of integrin inhibitors to the upregulated cell spreading on phosphorylated fibronectin on flat substrates. **A**: Cell spreading assay on fibronectin (Fn) fragments as defined in Fig 2. **B**: Inhibition of αvβ3 and α5β1 integrins on CKII-treated fibronectin. Fibroblasts were preincubated for 10 min with 2.5μg/ml integrin isoform-specific anti-αvβ3 antibodies, anti-α5 antibodies, or 10μg/ml cyclic RGD. Cell spreading area of single cells was measured 30 min after seeding. Statistical comparison done with Student’s *t*-test between pair of samples connected with lines on the column plots: ***, p-value<0.005; **, p-value<0.01; *, p-value<0.05; not significant (ns).

The cell spreading area was drastically decreased on the 40 kDa fragment compared to the native full-length fibronectin (Fig 4A). The lack of the prominent RGD-synergy binding site may explain such a low fibroblast spreading area. Treatment of the surface-bound 40 kDa fragment with CKII kinase or AP did not affect the cell spreading area, indicating that phosphorylation within these fibronectin domains is likely not responsible for the upregulated cell response observed with phosphorylated full-length fibronectin.

Similarly, phosphorylation of the 70 kDa fragment did not significantly affect the fibroblast spreading area, even though it contains numerous phosphorylation sites (Fig 4A). This supports the notion that the observed effects are not a nonspecific charge effect, but rather a specific effect of the phosphorylation itself. The fact that spreading on the 70 kDa fragments is rather high could be due to the denser and more accessible iso-DGR peptide recognized by αvβ3 integrins.

The mean cell spreading area on the 120 kDa fragment was in the same range as for full-length Fn (Fig 4A). Moreover, CKII-treatment of both proteins, the 120 kDa fragment and full-length fibronectin, enhanced the cell spreading area compared to the native samples by 12% for the 120 kDa fragment and 22% for full-length fibronectin, respectively (Fig 4A). In contrast, AP-treatment did not significantly alter the cell spreading area. These results suggest that phosphorylation sites located within the 120 kDa fragment contribute in distinct ways to the upregulated response of fibroblasts to fibronectin phosphorylation.

Next, we asked how soluble integrin inhibitors affect the spreading behavior. On phosphorylated full-length fibronectin (Fn-CKII) and in the presence of cyclic RGD (cRGD) peptides, the mean spreading area decreased by 60% (Fig 4B). In contrast, after blocking αvβ3 integrins with the LM609 antibody, which is known to bind to the βA domain of the β3 subunit, the spreading area decreased by approximately 10% compared to the control, whereas the blocking of α5β1 integrins by the JBS5 antibody, which binds to the β-propeller of the α5 subunit showed no significant changes. Simultaneous blocking of both types of integrins caused a ~20% decrease in the cell spreading area. These results suggest that the enhanced cell spreading is only partly mediated through specific integrin binding involving subunits β3 or, to a lesser extent, α5.

### Phosphorylation sites are evolutionarily conserved in fibronectin and concentrated around the RGD-binding site in other extracellular proteins

**Fig 5:**
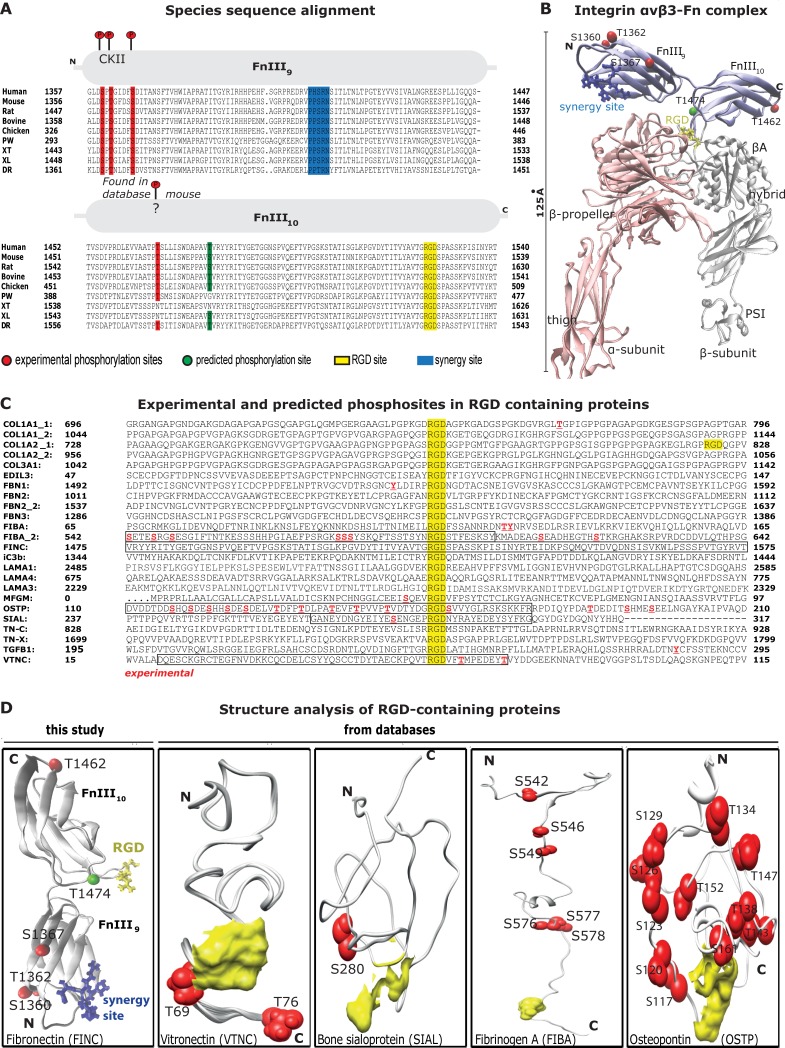
Putative phosphorylation sites around the RGD-binding site are highly conserved. **A:** Sequence alignment of the FnIII_9-10_ for various species, namely human, mouse, rat, bovine, chicken, pleurodeles waltl (PW), xenopus tropicalis (XT), xenopus laevis (XL) and danio rerio (DR). Experimentally determined phosphorylation sites for human fibronectin are depicted in red, predicted phosphorylation sites are shown in green. **B:** Structure of αvβ3 integrin headpiece docked to FnIII_9-10_. RGD (yellow) and synergy site (blue) are shown in sticks representation. **C:** Sequences of 20 RGD (yellow) containing human integrin ligands were analyzed -/+ 50 residues around their RGD. Shown are experimentally verified phosphor-sites. Black boxes indicate the sequences of the analyzed structures in D. **D:** The structures of fibronectin, vitronectin, bone sialoprotein, fibrinogen A and osteopontin were analyzed in proximity of their RGD (yellow) sites and experimentally verified phosphorylated sites (red) are highlighted.

If phosphorylation sites on ECM proteins are of physiological importance and regulate integrin binding, we next asked whether they are evolutionarily conserved. To this end, we first performed a sequence alignment comparing the integrin binding region FnIII_9_-FnIII_10_ in fibronectin among different species including human, mouse, rat, bovine and chicken, as well as the amphibians pleurodeles waltl, xenopus tropicalis, xenopus laevis and the zebrafish danio rerio. Our analysis revealed highly conserved phosphorylation sites within the integrin binding domains FnIII_9_-FnIII_10_ that are in spatial proximity to the RGD-loop in the folded domains (Fig 5A and 5B). Secondly, we analyzed other RGD-containing ECM proteins with respect to possible phosphorylation sites near the integrin binding RGD-site. Sequence analysis revealed that several experimentally reported phosphorylation sites retrieved from mass spectrometry data were also located close to the RGD binding site (Fig 5C), especially in the adhesion proteins osteopontin, fibrinogen A, vitronectin and bone sialoprotein. Similar to fibronectin, visualization of their secondary structures showed their close spatial proximity to the RGD binding loop (Fig 5D).

## Discussion

Although the phosphorylation of ECM proteins was reported already in 1883 [16] and in 1988 for fibronectin [8], we report here for the first time that the phosphorylation of fibronectin upregulates fundamental cell functions including cell spreading and proliferation (Fig 1) and leads to an increase of cell traction forces (Fig 2). Moreover, mass spectrometry was used to map the phosphorylated sites of human plasma fibronectin. We found a phosphorylated site S2384 on native fibronectin isolated from human blood near its C-terminus (Fig 3B). S2384 is located within an S-x-S motif that presumably becomes phosphorylated by the secreted kinase Fam20C [14]; 24 additional residues were phosphorylated by CKII (Fig 3B). Using proteolytic fibronectin fragments, generated by proteases found in blood, i.e. cathepsin D or α-chymotrypsin[17–20]. We identified that phosphorylation of residues located within the central 120 kDa fragment, containing the RGD-synergy binding sites, contribute to the effects of fibronectin phosphorylation on cell spreading (Fig 4A). Integrin inhibition, however, reduced but did not abolish these effects (Fig 4B), suggesting that integrins are involved in the observed upregulation of these processes, although they seem not to be the only driver. To which extent other cell surface receptors such as growth factor receptors synergistically contribute or cooperate with integrins in mediating the effects of phosphorylated fibronectin on cell function, requires further clarification.

Our observation that the phosphorylation of fibronectin affects cell spreading is in agreement with similar observations made for other phosphorylated ECM proteins including vitronectin [21], laminin-1 and osteopontin [22]. Whereas the phosphorylation of osteopontin was reported to enhance osteoclast migration, the phosphorylation of fibronectin by CKII did not induce a major change in the migratory behavior of fibroblasts in our assay (Figure B in S1 File). Our key finding is that the phosphorylation of fibronectin can upregulate local cell traction forces and the total strain energy per cell, which has never been reported before for any ECM protein. Previous studies had only focused on asking how intracellular phosphorylation can affect cell traction forces [23]. Cell traction forces are known to upregulate many intracellular processes, including the maturation of focal adhesions, as well as affect nuclear morphology and protein transcription profiles, and ultimately change various biological processes, including stem cell differentiation, cancer malignancy and development (for reviews see [24–26]).

Whether the phosphorylation of ECM proteins occurs during the secretory pathway and/or in the extracellular environment is currently a topic of debate [27, 28]. Clear evidence exists that some proteins might become phosphorylated already in the Golgi system prior to secretion [4, 29], for example by the kinases Fam20C [5] and VLK [4], as reported for osteopontin and laminin among many other proteins. As shown here, S2384 may be one such site on fibronectin that seems to become phosphorylated by Fam20C kinase prior secretion into the blood of healthy adult humans (Fig 3B and 3D).

Protein phosphorylation may also take place in the extracellular environment in situations where extracellular ATP concentrations transiently reach sufficiently high levels through the release of intracellular content. This can occur under various physiological and pathological conditions for example in the process of exocytosis of secretory vesicles, upon mechano-stimulation and under osmotic stress, when cells undergo necrosis or become damaged, as in wound sites and upon platelet activation [1]. While intracellular ATP levels range from 1 mM to 10 mM, extracellular concentrations can reach 40 μM in wound sites [30], while circulating plasma can contain ATP levels as high as 100 nM [31]. However, some ecto-kinases do not require the high ATP levels to operate optimally: protein kinase C (PKC) for example displays the highest activity at an ATP concentration of 100–500 nM [31]. Even a transient exposure of extracellular domains to ATP and kinases released by a damaged cell should thus be sufficient to lead to their phosphorylation and alter locally the biochemical signature of the ECM. Whether intracellular components, including protein kinases and ATP, are actively secreted to regulate physiological functions or are released due to cell death is currently under discussion [27, 28, 32]. Various studies have confirmed the regulated secretion of ATP into the extracellular environment [1] and kinase activity in extracellular environments, including that of CKII [33–36], protein kinase A (PKA) [27, 28, 37, 38], PKC [39–41]. In prostate cancer, PKA, PKC, and CKII are overexpressed in secretory vesicles and consequently released into the extracellular environment, supporting a physiological function of extracellular phosphorylation [27]. Moreover, a 10-fold increase in the serum levels of ecto-PKC in renal, colon, rectal, adrenal and lung cancer patients compared to normal serum has been reported [6, 32]. Furthermore, the serum levels of ecto-PKA kinase in melanoma patients positively correlates with the appearance and size of the tumor, while tumor removal reduced the level of ecto-PKA [28]. Indeed, inhibition of vesicle exocytosis was shown to inhibit glioma invasion, suggesting that the vesicle contents, including among others ATP and protein kinases, might play a major role in cancer progression and malignancy [42].

Here we show that phosphorylation of fibronectin increases the forces applied by fibroblasts to the perinuclear nanopillar adhesions (Fig 2C). As shown previously, perinuclear adhesions are rich in α5β1 integrins, and higher perinuclear tensile forces upregulate nuclear translocation of the transcription factor YAP [13]. Phosphorylation of fibronectin by CKII increased also the peripheral pillar displacements albeit to a lesser extent (Fig 2C). Cell spreading is dominated by the peripheral traction forces that cell can generate and at the onset of spreading the peripheral adhesions are still rich in both α5β1 and αvβ3 integrins, which might explain our observation that blocking either integrin on CKII-phosphorylated fibronectin had little effect on cell spreading compared to the native control. Only the blocking of both integrins, as expected, decreased the spreading of fibroblasts.

The importance of post-translational modifications of ECM proteins, including cross-linking, oxidation, hydroxylation, isomerization and glycosylation, has been highlighted for the regulation of cell-ECM interactions, especially in cancer progression [43]. However, possible effects of the phosphorylation of secreted proteins on the regulation of cell traction forces have mostly not been considered when asking how the biochemical signature of the ECM might affect cancer cells and their malignancy. The fact that the majority of phosphorylated sites in fibronectin have been found in cancer tissue samples in earlier studies suggests a potential role that needs to be further studied in the future [2]. Such findings might also apply to other diseases where enhanced kinase secretion might occur, and thus might have broad biological and medical consequences. While our analysis here focused on fibronectin, the phosphorylation of other ECM proteins [1, 22, 44] might cause similar effects or act synergistically to that of fibronectin. Indeed, a comparison of experimentally verified phosphorylation sites for various RGD-containing ECM proteins with their spatial relationship to the integrin binding site seems to support such a hypothesis (Fig 5C&5D). Insights into the importance of the phosphorylation state of ECM proteins on cell traction forces might also be used in the future for drug development. Possible effects of ECM phosphorylation on cell contractility and cell-ECM interactions have been largely overlooked when asking how the microenvironmental niches might regulate physiological or pathological processes. Whether the phosphorylation of fibronectin and of other ECM proteins in general is physiologically relevant or contributes to diseases such as cancer still needs to be determined. We hope that our data exploring how the controlled phosphorylation of fibronectin affects diverse cell functions will intensify the research on the physiological consequences of the phosphorylation of ECM proteins and will open up new directions and strategies for translational approaches.

## Materials and methods

### Bioinformatics

The bioinformatics prediction servers NetPhos 2.0 and NetPhosK 1.0 were used to predict putative phosphorylation sites with default settings. The phosphorylation data bank Phosida, PhosphoSitePlus, PhosphoNet, HPRD, dbPTM and the UniProt data bank were used to retrieve all experimentally verified phosphorylation sites for fibronectin. For, fibronectin, the Uniprot sequence accession number P02751-9 was used.

### Experimental

Human plasma fibronectin was purified in our lab using freshly pooled human plasma (see Section A in S1 File). 8-well Lab-tek chambers were coated with 20μg/ml diluted fibronectin over night at 4°C or 1h at room temperature and treated as described in the Section A in S1 File. Fibroblasts Fn -/- cells were cultured for three days in DMEM 4.5 μg/ml Glucose plus 10% serum until confluence for all experiments. Before treating with 0.025% trypsin for 3 min they were incubated for 10min in PBS at 37°C for gentle removal from the surface. The cells were centrifuged for 5 min at 5000U/min in serum-free fibroblast growth medium (Promocell G23010) and resuspended in fresh serum-free fibroblast growth medium for all experiments. Cells were diluted in fresh serum-free fibroblast growth medium to 100`000 cells/ml and used for the different cell experiment. Details about the assays used to quantify cell spreading, proliferation, migration and of the metabolic activity can be found in the Section A in S1 File, as well as our integrin blocking experiments.

### Metabolic activity assay

The colorimetric *WST*-*1* Cell Proliferation Assay from Roche (Cat. No. 11644807001) was used to measure metabolic cell activity. It is based on the cleavage of a tetrazolium salt by mitochondrial dehydrogenases to form formazan in viable cells. Cells were first incubated for 24h in 4-well Labtek chambers at 37°C in a cell incubator, followed by the addition of 50μl WST-1 reagent was to each well. Cells were again incubated for 1h at 37°C in the incubator. The medium was filtered and the absorbance was measured at A_450nm_-A_690nm_ according to the protocol. The mean values of 5 independent measurements were plotted. We used cell-free cell culture medium plus cell proliferation reagent WST-1 as a control to measure the background absorbance.

### Mass spectrometry

Human plasma fibronectin was either phosphorylated in solution following the supplier`s protocol with a CKII kinase (human recombinant E.coli, Calbiochem rhCKII (MERCK)) and then digested or first digested then phosphorylated and finally analyzed by mass spectrometry (MS) (Table A in S1 File).

### Nanopillar arrays for traction force measurements

Nanopillars were fabricated from the polymeric photoresistant SU-8 (MicroChem) as described previously [13]. Briefly, silicon nanopillar arrays were first fabricated by nanosphere lithography followed by plasma etching. Polydimethylsiloxane (PDMS) was then used to replicate the inverse structure, and SU-8 polymeric nanopillars were obtained by filling SU-8 liquid into PDMS molds, followed by hardening through UV exposure. The nanopillar arrays were placed into 30 mm glass-bottom MatTek dish (35mm) for cell culture. The nanopillars are 250 nm in diameter, 1.5 μm in height, with a periodicity of 800 nm. The spring constant was calibrated by measuring force-distance curves using atomic force microscopy to be 79 nN/μm. The nanopillar arrays were coated with human plasma fibronectin. Cells were then seeded on the nanopillars and the data shown here were taken 30 min after seeding. For time-lapse experiments, fibroblasts (10^4^ cells per mL) labeled with Vybrant Dil (1:200) (Invitrogen) were seeded on the nanopillar arrays and the imaging began 30 min after seeding. The displacements in xy-direction of the nanopillar tips were quantified by comparison of two images taken at the planes of the pillar tops versus the bottom plane. The images were then processed by particle tracking software (DiaTrack 3.03) to calculate pillar displacements (d). The corresponding traction forces (F) calculated according to Hooke’s law: F = k*d, where k represents the previously determined spring constant of the nanopillars, 79 nN/μm. Finally, strain energy (U) was calculated according to the formula: U = ½*k*d^2^. During image processing, cells were kept under constant conditions (37°C and 10% CO_2_). To calculate the traction forces exerted by cells on the nanopillar substrates, a Leica confocal microscope SP5 (63x, oil immersion, NA1.43) was used to measure the displacement of the nanopillar tips. Mean pillar displacements of three independent cells in three independent samples per each condition overtime were taken, whereby each time point corresponds to analyzing approximately 300–500 pillars underneath a cell after cell seeding. Each condition was imaged at seeding time point and after 30 minutes.

### Cell adhesion and spreading assay

200μl of diluted cells were added to each well. Unless stated otherwise, cells were allowed to adhere and spread for 30min. Medium was removed and cells were fixed with 2.5% formaldehyde in PBS for 30min at room temperature. Samples were washed several times with PBS and either directly analyzed or stored at 4°C. For the cell adhesion assays 5 random fields of view (80 x 80 μm) were recorded in two independent samples and for the cell spreading assays 50 randomly selected cells in duplicates, with no contact to other cells, were recorded using a confocal microscope in the middle of the well in two independent experiments. ImageJ was used to calculate the mean cell number and the mean cell spreading area.

### Fibronectin purification

Fibronectin was isolated from fresh human blood that was obtained from Zürcher Blutspendedienst (SRK) using gelatin-sepharose chromatography according to our established protocols. Blood of two independent patients was first pooled. Briefly, the mixture of 0.01 M EDTA, 0.002 M phenylmethyl- sulphonyl fluoride and the human plasma were spun at 15,000 g for 40 min. The plasma was the passed over a Sepharose 4B column (Pharmacia) and Sepharose 4B column (Sigma-Aldrich). Afterwards the gelatin column was washed with 0.002 M phenylmethyl- sulphonyl fluoride and 0.01 M EDTA in PBS. The column was again washed with 1 M NaCl and 1 M urea. Finally the product was eluted with 6M urea. The purity was confirmed by silver stain and western blot. Isolated fibronectin was stored at − 80°C in 6 M urea until usage. The 70 kDa fibronectin fragment was generated with cathepsin D, and the 10 kDa and 40 kDa fragments with alpha-chymotrypsin. Both these proteases can be found in serum and presumably can cleave FN[17–20].

### Lab-tek chamber treatment

The background was blocked with 200μl 2% BSA in PBS for 30min. Samples were washed 3X with PBS. CKII kinase (human recombinant E.coli, Calbiochem rhCKII (MERCK) diluted 1:100 in PBS), Alkaline phosphatase (Roche alkaline phosphatase from calf intestine no.10.713023.001, 1unit/μl), control solutions were prepared. AP (dephosphorylated): 200μl H20; 200μl 2x buffer (0,05M TRIS, pH 7.5; 0,15M NaCl; 0,01M MgCl2); 2μl ATP (0,1M), 2μl alkaline phosphtase (AP). NA (native): 200μl H20; 200μl 2x buffer, 2μl ATP. CKII (hyper-phosphorylated): 200μl H20, 200μl 2x buffer (0,05M TRIS, pH 7.5; 0,15M NaCl; 0,01M MgCl2); 2μl ATP; 2μl casein kinase II (CKII). 200μl solution was added to the appropriate samples. Samples were incubated for 2.5h at 37°C in a cell incubator. Samples were washed 3X with PBS and either directly used or stored in PBS at 4°C.

### Integrin blocking experiments

The cRGD (cyclo (-RGDFC) cat. No. 63786–1, ANASPEC) was diluted to 10μg/ml in the 200μl cell samples. The anti-αvβ3 antibody (clone LM6 Fisher-Scientific MAB1976BMI) was diluted to 2.5μg/ml in the 200μl cell samples. And the anti-α5 antibody (abcam, ab23589, JBS5) was diluted to 2.5μg/ml in the 200μl cell samples. Cells were preincubated with the antibodies or cRGD peptides for 10min before seeding. The mean relative spreading area of 50 randomly selected single cells are compared under different conditions) on CKII-treated Fibronectin. As a control we used Fn-CKII without the antibodies.

### Confocal microscopy and image analysis

The mean of the spreading area of 50 randomly selected single cells in the middle of the well per sample were analyzed in duplicates in two independent experiments. In order to further minimize the error, two independently coated wells were merged to one sample. Thereby 25 cells from each well were analyzed.

## Supporting information

S1 Dataset Minimal data set. (ZIP)

S1 File Section A. **Materials and Methods. Figure A. Effect of fibronectin phosphorylation on cell spreading.** Cells were allowed to adhere and spread for 30min. Medium was removed, and cells were fixed with 2.5% formaldehyde in PBS for 30min at room temperature for the different conditions. For the cell spreading assays 50 randomly selected cells in duplicates, with no contact to other cells, were recorded using a confocal microscope in the middle of the well in two independent experiments. Ten randomly picked cells are shown for each of the conditions using phase contrast. The magnification is the same for all cells shown. **Figure B. Effect of fibronectin phosphorylation on cell migration and proliferation on flat fibronectin-coated surfaces. A**: Cell migration analysis 60 min. after seeding. The migration behavior was analyzed with regards to the total travelled path (a), the Euclidian start-to-end distance (b), the average velocity (c), the relative directionality (d) analyzing 38, 39 and 22 cells respectively indicated by lines in different colors. **B**: Cell proliferation analysis after 24h and 48h. Prior to the assay, Fn-/- fibroblasts and controls were synchronized in this experiment only using the standard aphidicolin cell synchronization protocol. The p-values have been calculated by 1-way ANOVA and the pairwise comparisons with the Tukey test. **Figure C. Phosphorylated sites identified by mass spectrometry and retrieved from data banks for other species.** The phosphorylation data banks Phosida, PhosphoSitePlus, PhosphoNet, dbPTM, HPRD and UniProt were searched for phosphorylated site for mouse, rat and bovine fibronectin. **Table A. Phosphorylated sites identified by mass spectrometry.** M: Mascot only S: Sequest only S*: no Mascot search performed prot = phosphorylation of protein followed by enzymatic digestion pep = phosphorylation of peptides after enzymatic digestions (after fragmentation) in blue are the hits that were only found by Mascot (in Mascot there is no separate criterium apart from the peptide score that tells us how localized the phosphorylation is). However, they were found with a high confidence (see filter criteria) and can be considered as relevant (if one trusts Mascot, as many people do). *filter settings*: included are only those results with pep score > 25 (Mascot) pep prob > 0.9 (Sequest) and Ascore > 15 (= localized by >90%) **Table B. Phosphorylated sites identified by mass spectrometry and software predication. A:** Phosphorylation sites as identified by mass spectrometry after phosphorylating human plasma fibronectin in solution by CKII or PKC. **B:** Phosphorylation sites as predicted using the server NetPhosK.
(DOCX)
