# Supplementary material for: Phosphorylated fibronectin enhances cell attachment and upregulates mechanical cell functions
Source: PLoS One. 2019 Jul 10;14(7):e0218893. doi: 10.1371/journal.pone.0218893 (PMC6619657; doi:10.1371/journal.pone.0218893)
Supplement: S1 File — Materials and Methods. Figure A. Effect of fibronectin phosphorylation on cell spreading. Cells were allowed to adhere and spread for 30min. Medium was removed, and cells were fixed with 2.5% formaldehyde in PBS for 30min at room temperature for the different conditions. For the cell spreading assays 50 randomly selected cells in duplicates, with no contact to other cells, were recorded using a confocal microscope in the middle of the well in two independent experiments. Ten randomly picked cells are shown for each of the conditions using phase contrast. The magnification is the same for all cells shown. Figure B. Effect of fibronectin phosphorylation on cell migration and proliferation on flat fibronectin-coated surfaces. A: Cell migration analysis 60 min. after seeding. The migration behavior was analyzed with regards to the total travelled path (a), the Euclidian start-to-end distance (b), the average velocity (c), the relative directionality (d) analyzing 38, 39 and 22 cells respectively indicated by lines in different colors. B: Cell proliferation analysis after 24h and 48h. Prior to the assay, Fn-/- fibroblasts and controls were synchronized in this experiment only using the standard aphidicolin cell synchronization protocol. The p-values have been calculated by 1-way ANOVA and the pairwise comparisons with the Tukey test. Figure C. Phosphorylated sites identified by mass spectrometry and retrieved from data banks for other species. The phosphorylation data banks Phosida, PhosphoSitePlus, PhosphoNet, dbPTM, HPRD and UniProt were searched for phosphorylated site for mouse, rat and bovine fibronectin. Table A. Phosphorylated sites identified by mass spectrometry. M: Mascot only S: Sequest only S*: no Mascot search performed prot = phosphorylation of protein followed by enzymatic digestion pep = phosphorylation of peptides after enzymatic digestions (after fragmentation) in blue are the hits that were only found by Mascot (in Mascot there is no separate criterium [file pone.0218893.s002.docx]

Supplementary Materials

Title

Phosphorylated fibronectin enhances cell grip and upregulates vital mechanical functions

**Authors**

Garif Yalak,^1^ Jau-Ye Shiu,^1^ Ingmar Schön, Maria Mitsi,^1^ Viola Vogel^1*^

**Affiliations**

^1^ Laboratory of Applied Mechanobiology, Institute of Translational Medicine, Department of Health Sciences and Technology, Swiss Federal Institute of Technology (ETH) Zurich, Vladimir-Prelog-Weg 4, ETH Zürich, HCI E415, 8093 Zürich

**Author affiliations:**

*Corresponding author:

Viola Vogel

^1^Laboratory of Applied Mechanobiology

Department of Health Sciences and Technology

ETH Zurich

Vladimir-Prelog-Weg 4, HCI F443

CH-8093 Zurich, Switzerland

Phone: +41 44 632 08 87
Fax: +41 44 632 10 73
E-Mail: viola.vogel@hest.ethz.ch

**Section A. Materials and Methods.**

***Bioinformatics***

The bioinformatics prediction servers NetPhos 2.0 [1] and NetPhosK 1.0 [2], using neuronal networks, were used to predict putative phosphorylation sites with default settings*.* The phosphorylation data bank Phosida [3] , PhosphoSitePlus [4] , PhosphoNet HPRD [5] dbPTM [6] and the UniProt data bank [7] were used to retrieve all experimental phosphorylation sites for fibronectin.

***Multiple sequence alignments***

Sequences were retrieved from the UniProt database. Multiple sequence alignments were calculated using ClustalW using default settings. The Jalview software package was used to visualize and analyze the multiple sequence alignment.

***Structural and phylogenetic analysis***

Following sequences were retrieved from the UniProt data [7] ([http://www.uniprot.org](http://www.uniprot.org/)). for Figure 6: COL1A1 (P02452), COL1A2 (P08123), COL3A1 (P02461), EDIL3 (O43854), FBN1 (P35555), FBN2 (P35556), FBN3 (Q75N90), FIBA (P02671), FINC (P02751), iC3b (P01024), LAMA1 (P25391), LAMA 3 (Q16787), LAMA4 (Q16363), MFGM (Q08431), OSTP (P10451), SIAL (P21815), TN-C (P24821), TN-X (P22105), TGFB1 (P01137) and VTNC (P04004). Multiple sequence alignments were calculated using ClustalW [8] (<http://www.ebi.ac.uk/Tools/msa/clustalw2>) using default settings. The Jalview [9] (<http://www.jalview.org/>) software package was used to visualize and analyze the multiple sequence alignment.

***Fibronectin purification***

Fibronectin was isolated from fresh human blood that was obtained from Zürcher Blutspendedienst (SRK) using gelatin-sepharose chromatography according to our established protocols. Blood of two independent patients was first pooled. Briefly, the mixture of 10 mM EDTA, 2 mM phenylmethyl- sulphonyl fluoride and the human plasma were spun at 15,000 g for 40 min. The plasma was the passed over a Sepharose 4B column (Pharmacia) and Sepharose 4B column (Sigma-Aldrich). Afterwards the gelatin column was washed with 2 mM phenylmethyl- sulphonyl fluoride and 10 mM EDTA in PBS. The column was again washed with 1 M NaCl and 1 M urea. Finally, the product was eluted with 6M urea. The purity was confirmed by silver stain and western blot. Isolated fibronectin was stored at − 80 °C in 6 M urea until usage.

***Mass spectrometry***

Fibronectin (Uniprot sequence accession number: P02751-9) was either phosphorylated in solution following the supplier`s protocol with a CKII kinase (human recombinant E.coli, Calbiochem rhCKII (MERCK)) or PKC kinase (PKC alpha, 0.10μg; PK-PKCA-A010, proteinkinase.de) and then digested or first digested then phosphorylated and finally analyzed by mass spectrometry. The mass spectrometry data were analyzed by using multi-stage activation and a probability based score [10]. The Ascore algorithm for phosphorylation site localization was used to assign a confidence value to each phosphorylation site by reanalyzes of the phosphopeptides. The standard programs Mascot and Sequest were used to analyze the MS data using the settings considered high confidence. The Mascot score was set to >25. The probability for Sequest was set to 0.9 and for Ascore to >15, which is a localization > 90%.

Fibronectin (Uniprot sequence accession number **P02751-9**) was either phosphorylated in solution following the supplier`s protocol with a CKII kinase (human recombinant E.coli, Calbiochem rhCKII (MERCK)) or PKC kinase (PKC alpha, 0.10μg; PK-PKCA-A010, proteinkinase.de) and then digested or first digested then phosphorylated and finally analyzed by mass spectrometry.

***Lab-tek chamber treatment***

The background was blocked with 200μl 2% BSA in PBS for 30min. Samples were washed 3X with PBS. CKII kinase (human recombinant E.coli, Calbiochem rhCKII (MERCK) diluted 1:100 in PBS), Alkaline phosphatase (Roche alkaline phosphatase from calf intestine no.10.713023.001, 1unit/μl), control solutions were prepared. AP (dephosphorylated): 200μl H20; 200μl 2x buffer (0,05M TRIS, pH 7.5; 0,15M NaCl; 0,01M MgCl2); 2μl ATP (0,1M), 2μl alkaline phosphtase (AP). NA (native): 200μl H20; 200μl 2x buffer, 2μl ATP. CKII (hyper-phosphorylated): 200μl H20, 200μl 2x buffer (0,05M TRIS, pH 7.5; 0,15M NaCl; 0,01M MgCl2); 2μl ATP; 2μl casein kinase II (CKII). 200μl solution was added to the appropriate samples. Samples were incubated for 2.5h at 37°C in a cell incubator. Samples were washed 3X with PBS and either directly used or stored in PBS at 4°C.

***Cell adhesion and spreading assay***

200μl of diluted cells were added to each well. Unless stated otherwise, cells were allowed to adhere and spread for 30min. Medium was removed and cells were fixed with 2.5% formaldehyde in PBS for 30min at room temperature. Samples were washed several times with PBS and either directly analyzed or stored at 4°C. For the cell adhesion assays 5 random fields of view (80 x 80 μm) were recorded in two independent samples and for the cell spreading assays 50 randomly selected cells in duplicates, with no contact to other cells, were recorded using a confocal microscope in the middle of the well in two independent experiments. ImageJ was used to calculate the mean cell number and the mean cell spreading area.

***Confocal microscopy and image analysis***

The mean of the spreading area of 50 randomly selected single cells in the middle of the well per sample were analyzed in duplicates in two independent experiments. In order to further minimize the error, two independently coated wells were merged to one sample. Thereby 25 cells from each well were analyzed.

***Integrin blocking experiments***

The cRGD (cyclo (-RGDFC) cat. No. 63786-1, ANASPEC) was diluted to 10μg/ml in the 200μl cell samples. The anti-αvβ3 antibody (clone LM6 Fisher-Scientific MAB1976BMI[11]) was diluted to 2.5μg/ml in the 200μl cell samples. And the anti-α5 antibody (abcam, ab23589, JBS5 [11]) was diluted to 2.5μg/ml in the 200μl cell samples. Cells were preincubated with the antibodies or cRGD peptides for 10min before seeding. The mean relative spreading area of 50 randomly selected single cells are compared under different conditions) on CKII-treated Fibronectin. As a control we used Fn-CKII without the antibodies.

Supplementary Materials Figures and Tables


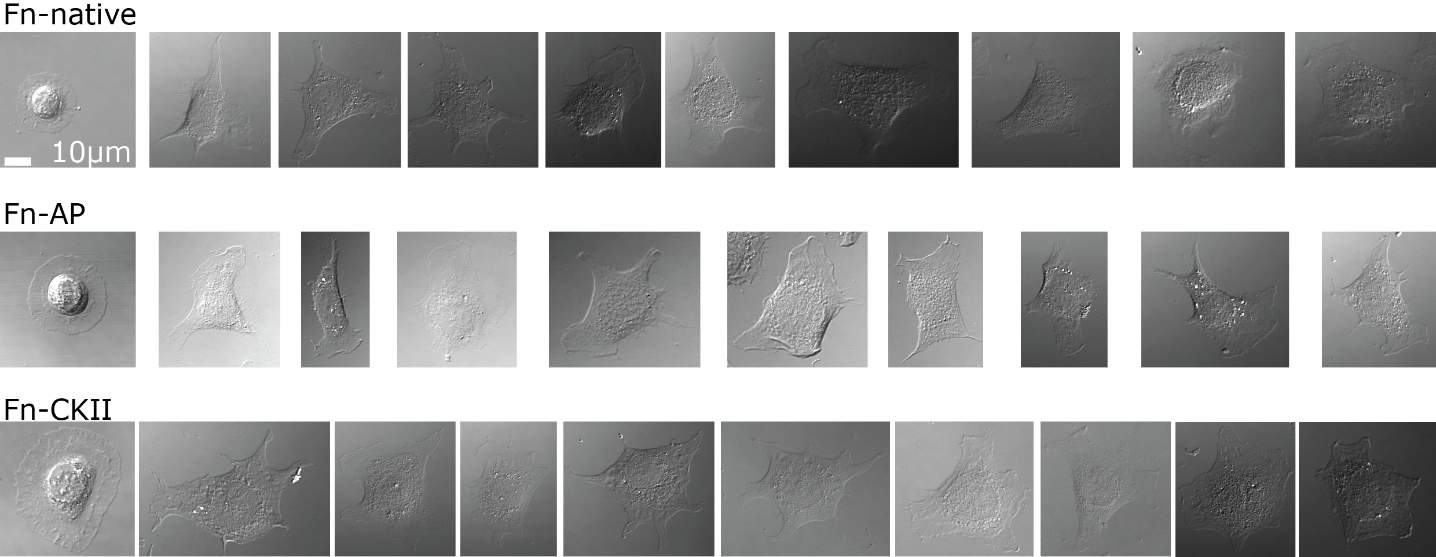


**Figure A. Effect of fibronectin phosphorylation on cell spreading.**

Cells were allowed to adhere and spread for 30min. Medium was removed, and cells were fixed with 2.5% formaldehyde in PBS for 30min at room temperature for the different conditions. For the cell spreading assays 50 randomly selected cells in duplicates, with no contact to other cells, were recorded using a confocal microscope in the middle of the well in two independent experiments. Ten randomly picked cells are shown for each of the conditions using phase contrast. The magnification is the same for all cells shown.

**
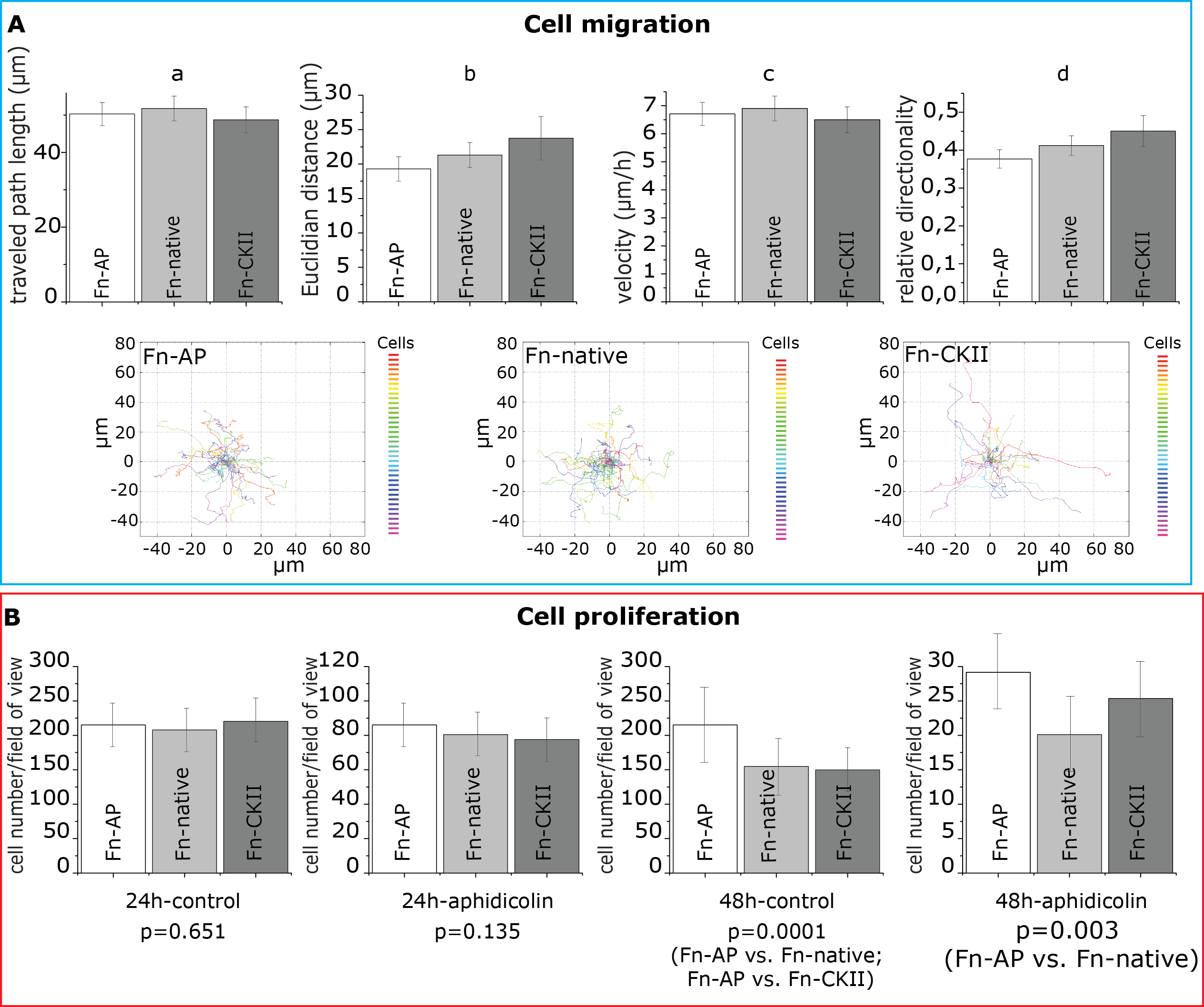
** **Figure B. Effect of fibronectin phosphorylation on cell migration and proliferation on flat fibronectin-coated surfaces. A**: Cell migration analysis 60 min. after seeding. The migration behavior was analyzed with regards to the total travelled path (a), the Euclidian start-to-end distance (b), the average velocity (c), the relative directionality (d) analyzing 38, 39 and 22 cells respectively indicated by lines in different colors. **B**: Cell proliferation analysis after 24h and 48h. Prior to the assay, Fn-/- fibroblasts or controls were synchronized using the standard aphidicolin cell synchronization protocol. The p-values have been calculated by 1-way ANOVA and the pairwise comparisons with the Tukey test.

**
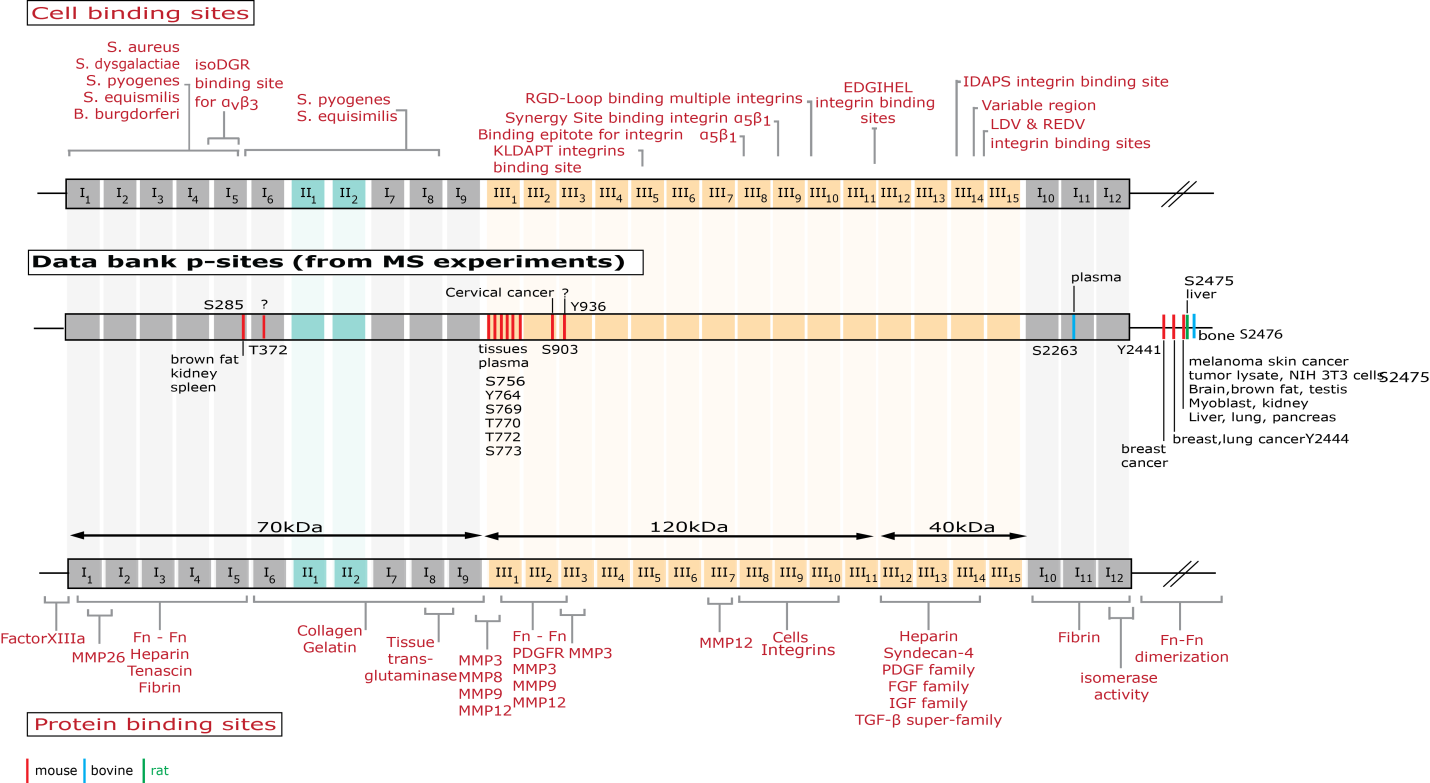
**

**Figure C. Phosphorylated sites identified by mass spectrometry and retrieved from data banks for other species.** The phosphorylation data banks Phosida, PhosphoSitePlus, PhosphoNet, dbPTM, HPRD and UniProt were searched for phosphorylated site for mouse, rat and bovine fibronectin. Proteolytic fragments, molecular recognition sites and cell binding sites are indicated [12-15].

**Table A. Phosphorylated sites identified by mass spectrometry.**

| **residue** | | **sequence** | **native** | **CKII prot** | **CKII pep** | **PKC prot** | **PKC pep** |
| --- | --- | --- | --- | --- | --- | --- | --- |
| T | 96 | EE**T**CF |  |  | 10-10-28  10-12-16 |  |  |
| T | 136 | SC**T**IA |  |  |  |  | 10-12-16 |
| T | 193 | AG**T**SY |  |  | 10-10-28M |  |  |
| T | 214 | DC**T**CL |  |  | 10-10-28S |  |  |
| S | 280 | TT**S**SG |  |  | 10-12-16M |  |  |
| T | 365 | PF**T**YN |  |  | 10-10-28M |  |  |
| S | 373 | FY**S**CT |  |  | 10-10-28  10-12-16M |  |  |
| S | 390 | SE**T**GT |  |  | 10-12-16M |  |  |
| T | 402 | FC**T**DH |  |  | 10-10-28M |  |  |
| T | 471 | IC**T**TN |  |  | 10-10-28M |  |  |
| T | 564 | QD**S**ET |  |  | 10-10-28 |  |  |
| T | 566 | SE**T**GT |  |  | 10-10-28M  10-12-16M |  |  |
| S | 757 | EL**S**EE |  |  | 10-10-28S |  |  |
| S | 852 | EG**S**ST |  |  | 10-10-28M |  |  |
| T | 1132 | EV**T**SD |  |  | 10-10-28M |  |  |
| T | 1192 | VL**T**VS |  |  | 10-12-16 |  |  |
| T | 1296 | DL**T**NF |  |  | 10-12-16S |  | 10-12-16 |
| T | 1356 | QK**T**GL |  |  | 10-07-15S* |  |  |
| S | 1360 | LD**S**PT |  |  | 10-07-15S* |  |  |
| T | 1362 | SP**T**GI |  |  | 10-07-15S* |  |  |
| S | 1367 | DF**S**DI |  |  | 10-10-28 |  |  |
| T | 1877 | PG**T**DY |  |  | 10-12-16 |  |  |
| T | 2141 | VG**T**DE |  |  | 10-10-28 |  |  |
| S | 2225 | RM**S**ES |  |  | 10-10-28 |  |  |
| T | 2294 | EA**T**CY |  |  | 10-10-28  10-12-16 |  |  |
| S | 2384 | ED**S**RE | 10-10-28 | 10-10-28  10-12-16 | 10-10-28  10-12-16 | 10-12-16 | 10-12-16 |

Numbering according to fibronectin isoform P02751-1

*legend:*

M: Mascot only

S: Sequest only

S*: no Mascot search performed

prot = phosphorylation of protein followed by enzymatic digestion

pep = phosphorylation of peptides after enzymatic digestions (after fragmentation)

in blue are the hits that were only found by Mascot (in Mascot there is no separate criterium apart from the peptide score that tells us how localized the phosphorylation is). However, they were found with a high confidence (see filter criteria) and can be considered as relevant (if one trusts Mascot, as many people do).

*filter settings:* included are only those results with

- pep score > 25 (Mascot)
- pep prob > 0.9 (Sequest) and Ascore > 15 (=localized by >90%)

**Table B. Phosphorylated sites identified by mass spectrometry and software predication.**

| 1. **Phosphorylated sites identified by mass spectrometry (this study)** | | | | | | | | | |
| --- | --- | --- | --- | --- | --- | --- | --- | --- | --- |
|  | | | | | | | | | |
| **Method** | **Residue** | **Kinase** | | **Uniprot- code** | **Location**  **Binding sites** | **Reference**  **Prediction**  **Datbases** | **Species** | **Tissue**  **Cells** |  |
| MS  phosphorylation of peptides (after fragmentation) | T96 | Casein kinase II (CKII) | | P02751 | FnI2  Fibrin, Heparin, Fn-Fn, Tenascin, S.aureus, S.pyogenes,  Fn-ase  binding | this study  NetPhos 2.0 | human | blood plasma |  |
| MS  phosphorylation of peptides (after fragmentation) | T136 | Protein kinase C (PKC) | | P02751 | FnI2  Fibrin, Heparin, Fn-Fn, Tenascin, S.aureus, S.pyogenes,  FN-ase  binding | this study  NetPhos 2.0  Experimentally proven:  Found in 1 patients sample  Jurkat  T cell leukemia | human | blood plasma |  |
| MS  phosphorylation of peptides (after fragmentation) | T193 | Casein kinase II (CKII) | | P02751 | FnI4  Fibrin, Heparin, Fn-Fn, Tenascin, S.aureus, S.pyogenes,  FN-ase  binding | this study  NetPhosK (PKC)  NetPhos 2.0 | human | blood plasma |  |
| MS  phosphorylation of peptides (after fragmentation) | T214 | Casein kinase II (CKII) | | P02751 | FnI4  Fibrin, Heparin, Fn-Fn, Tenascin, S.aureus, S.pyogenes,  Fn-ase  binding | this study  NetPhos 2.0 | human | blood plasma |  |
| MS  phosphorylation of peptides (after fragmentation) | S280 | Casein kinase II (CKII) | | P02751 | Linker FnI5-Fn6 | this study  NetPhos 2.0 | human | blood plasma |  |
| MS  phosphorylation of peptides (after fragmentation) | T365 | Casein kinase II (CKII) | | P02751 | FnII1  Collagen, Iso-DGR for αvβ3, Gelatine, FnColA-ase,  FnColB-ase  binding | this study  NetPhosK (PKC)  NetPhos 2.0 | human | blood plasma |  |
| MS  phosphorylation of peptides (after fragmentation) | S373 | Casein kinase II (CKII) | | P02751 | FnII1  Collagen, Iso-DGR for αvβ3, Gelatine, FnColA-ase,  FnColB-ase  binding | this study  NetPhos 2.0 | human | blood plasma |  |
| MS  phosphorylation of peptides (after fragmentation) | S390 | Casein kinase II (CKII) | | P02751 | FnII1  Collagen, Iso-DGR for αvβ3, Gelatine, FnColA-ase,  FnColB-ase  binding | this study  NetPhosK (CKII)  NetPhos 2.0 | human | blood plasma |  |
| MS  phosphorylation of peptides (after fragmentation) | T402 | Casein kinase II (CKII) | | P02751 | FnII1  Collagen, Iso-DGR for αvβ3, Gelatine, FnColA-ase,  FnColB-ase  binding | this study  NetPhos 2.0 | human | blood plasma |  |
| MS  phosphorylation of peptides (after fragmentation) | T471 | Casein kinase II (CKII) | | P02751 | FnI7  Collagen ,Gelatine  FnColA-ase,  FnColB-ase  binding | this study  NetPhosK (CKII)  NetPhos 2.0 | human | blood plasma |  |
| MS  phosphorylation of peptides (after fragmentation) | T564 | Casein kinase II (CKII) | | P02751 | FnI9 | this study  NetPhosK (CKI)  NetPhos 2.0 | human | blood plasma |  |
| MS  phosphorylation of peptides (after fragmentation) | T566 | Casein kinase II (CKII) | | P02751 | FnI9 | this study  NetPhos 2.0 | human | blood plasma |  |
| MS  phosphorylation of peptides (after fragmentation) | S757 | Casein kinase II (CKII)S-x-E | | P02751 | FnIII2  Fn-Fn  binding | this study  NetPhosK (CKI and CKII)  NetPhos 2.0 | human | blood plasma |  |
| MS  phosphorylation of peptides (after fragmentation) | S852 | Casein kinase II (CKII) | | P02751 | FnIII3 | this study  NetPhosK (CKII)  NetPhos 2.0 | human | blood plasma |  |
| MS  phosphorylation of peptides (after fragmentation) | T1132 | Casein kinase II (CKII) | | P02751 | FnIII6  DNA binding | this study  NetPhos 2.0 | human | blood plasma |  |
| MS  phosphorylation of peptides (after fragmentation) | T1192 | Casein kinase II (CKII) | | P02751 | FnIII7  Cryptic cysteine  Fn binding | this study  NetPhos 2.0 | human | blood plasma |  |
| MS  phosphorylation of peptides (after fragmentation) | T1296 | Casein kinase II (CKII)  Protein kinase C (PKC) | | P02751 | FnIII8  Binding epitope for α5β1  Cell attachment | this study  NetPhos 2.0 | human | blood plasma |  |
| MS  phosphorylation of peptides (after fragmentation) | T1356 | Casein kinase II (CKII) | | P02751 | FnIII8  Binding epitope for α5β1  Cell attachment | this study  NetPhosK (PKB)  NetPhos 2.0 | human | blood plasma |  |
| MS  phosphorylation of peptides (after fragmentation) | S1360 | Casein kinase II (CKII) | | P02751 | FnIII9  Synergy site binding integrin α5β1  Fn-Fn binding  Cell attachement | this study  NetPhosK (GSK3)  NetPhos 2.0 | human | blood plasma |  |
| MS  phosphorylation of peptides (after fragmentation) | T1362 | Casein kinase II (CKII) | | P02751 | FnIII9  Synergy site binding integrin α5β1  Fn-Fn  Cell attachement | this study  NetPhos 2.0 | human | blood plasma |  |
| MS  phosphorylation of peptides (after fragmentation) | S1367 | Casein kinase II (CKII) | | P02751 | FnIII9  Synergy site binding integrin α5β1  Fn-Fn binding  Cell attachement | this study  NetPhos 2.0 | human | blood plasma |  |
| MS  phosphorylation of peptides (after fragmentation) | T1877 | Casein kinase II (CKII) | | P02751 | FnIII13  Heparin,  Syndecan-4,  Fn binding | this study  NetPhosK (cdc2)  NetPhos 2.0 | human | blood plasma |  |
| MS  phosphorylation of peptides (after fragmentation) | T2141 | Casein kinase II (CKII) | | P02751 | Variable IIICS  LDV & REDV integrin binding sites | this study  NetPhosK (CKII)  NetPhos 2.0 | human | blood plasma |  |
| MS  phosphorylation of peptides (after fragmentation) | S2225 | Casein kinase II (CKII) | | P02751 | Variable IIICS  LDV & REDV integrin binding sites | this study  NetPhosK (CKI)  NetPhos 2.0 | human | blood plasma |  |
| MS  phosphorylation of peptides (after fragmentation) | T2294 | Casein kinase II (CKII) | | P02751 | FnIII15  Cryptic cysteine  Fn binding | this study  NetPhos 2.0 | human | blood plasma |  |
| MS  phosphorylation of peptides (after fragmentation)  phosphorylation of protein, then digestion | S2384 | **Native**  Casein kinase II (CKII), very likely of native origin  Protein kinase C (PKC), very likely of native origin | | P02751 | FnI10  Fibrin-binding | this study  NetPhosK (RSK)  NetPhos 2.0  S2238 (corresponds to S2384 in Fn isoform P02751-1)  Phosida  PhosphoSitePlus  PhosphoNet  dbPTM  UniProt  HPRD | human | blood plasma |  |
| **B) Phosphorylation sites as predicted using the server NetPhosK 3.1** | | | | | | | | | |
| **Method** | **Residue** | | **Kinase** | **Uniprot- code** | **Location/binding sites** | **Reference**  **Prediction**  **Datbases** | **Species** | **Tissue**  **Cells** |  |
| Predicted | S-89 | | Casein kinase II (CKII) | P02751 | FnI1  Fibrin, Heparin, Fn-Fn, Tenascin, S.aureus, S.pyogenes,  Fn-ase | NetPhosK, NetPhos 2.0 | human | / |  |
| Predicted | T-287 | | Casein kinase II (CKII) | P02751 | Loop FnI5-FnI6 | NetPhosK, NetPhos 2.0 | human | / |  |
| Predicted | S-390 | | Casein kinase II (CKII) | P02751 | FnII1  Collagen binding  Iso-DGR for αvβ3  Gelatine binding  FnColA-ase  FnColB-ase | NetPhosK, NetPhos 2.0, | human | / |  |
| Predicted | T-432 | | Casein kinase II (CKII) | P02751 | FnII2  Collagen binding  Gelatine binding  FnColA-ase  FnColB-ase | NetPhosK | human | / |  |
| Predicted | T-471 | | Casein kinase II (CKII) | P02751 | FnI7  Collagen binding  Gelatine binding  FnColA-ase  FnColB-ase | NetPhosK, NetPhos 2.0, | human | / |  |
| Predicted | S-727 | | Casein kinase II (CKII) | P02751 | FnIII2  Fn-Fn  FnI1-5 | NetPhosK, NetPhos 2.0, | human | / |  |
| Predicted | T-729 | | Casein kinase II (CKII) | P02751 | FnIII2  Fn-Fn  FnI1-5 | NetPhosK, NetPhos 2.0, | human | / |  |
| Predicted | S-757 | | Casein kinase II (CKII) | P02751 | FnIII2  Fn-Fn  FnI1-5 | NetPhosK, NetPhos 2.0 | human | / |  |
| Predicted | S-794 | | Casein kinase II (CKII) | P02751 | FnIII2  Fn-Fn  FnI1-5 | NetPhosK, NetPhos 2.0 | human | / |  |
| Predicted | S-852 | | Casein kinase II (CKII) | P02751 | FnIII3 | NetPhosK, NetPhos 2.0, | human | / |  |
| Predicted | T-854 | | Casein kinase II (CKII) | P02751 | FnIII3 | NetPhosK, NetPhos 2.0 | human | / |  |
| Predicted | T-1096 | | Casein kinase II (CKII) | P02751 | FnIII6  DNA binding | NetPhosK, NetPhos 2.0 | human | / |  |
| Predicted | S-1220 | | Casein kinase II (CKII)  S-x-E | P02751 | FnIII7  Cryptic cysteine  FnIII1 exposed by FnIIIEDB | NetPhosK, NetPhos 2.0, | human | / |  |
| Predicted | S-1340 | | Casein kinase II (CKII) | P02751 | FnIII8  Binding epitope for α5β1  Cell attachement | NetPhosK, NetPhos 2.0, | human | / |  |
| Predicted | S-1667 | | Casein kinase II (CKII) | P02751 | EDA | NetPhosK, NetPhos 2.0, | human | / |  |
| Predicted | T-1686 | | Casein kinase II (CKII) | P02751 | EDA | NetPhosK, NetPhos 2.0 | human | / |  |
| Predicted | T-1809 | | Casein kinase II  (CKII) | P02751 | FnIII12  Heparin  Syndecan-4  FnIII1 | NetPhosK, NetPhos 2.0, | human | / |  |
| Predicted | T-1961 | | Casein kinase II (CKII) | P02751 | FnIII14  Heparin  Syndecan-4  FnIII1  Anti-adhesive site | NetPhosK, NetPhos 2.0 | human | / |  |
| Predicted | T-2141 | | Casein kinase II (CKII) | P02751 | FnIII15  Cryptic cysteine  FnIII1 | NetPhosK, NetPhos 2.0 | human | / |  |
| Predicted | T-2202 | | Casein kinase II (CKII) | P02751 | Loop FnIII15-FnI10  Fibrin | NetPhosK, NetPhos 2.0 | human | / |  |

**A:** Phosphorylation sites as identified by mass spectrometry after phosphorylating human plasma Fn in solution by CKII or PKC. **B:** Phosphorylation sites as predicted using the server NetPhosK.

**References Supplements**

[1] N. Blom, S. Gammeltoft, S. Brunak, Sequence- and structure-based prediction of eukaryotic protein phosphorylation sites., Journal of Molecular Biology 294((5)) (1999) 1351-1362.

[2] N. Blom, T. Sicheritz-Ponten, R. Gupta, S. Gammeltoft, S. Brunak, Prediction of post-translational glycosylation and phosphorylation of proteins from the amino acid sequence Proteomics 4((6)) (2004 ) 1633-49.

[3] F. Gnad, S. Ren, J. Cox, J. Olsen, B. Macek, M. Oroshi, M. Mann, PHOSIDA (phosphorylation site database): management, structural and evolutionary investigation, and prediction of phosphosites., Genome Biol. 8(11)((11):R250.) (2007).

[4] V.P. Hornbeck, J.M. Kornhauser, S. Tkachev, B. Zhang, E. Skrzypek, B. Murray, V. Latham, M. Sullivan, PhosphoSitePlus: a comprehensive resource for investigating the structure and function of experimentally determined post-translational modifications in man and mouse, Nucleic Acids Research 40(D1) (2011) D261-D270.

[5] T. Keshava Prasad, R. Goel, K. Kandasamy, S. Keerthikumar, S. Kumar, S. Mathivanan, D. Telikicherla, R. Raju, B. Shafreen, A. Venugopal, L. Balakrishnan, A. Marimuthu, S. Banerjee, D. Somanathan, A. Sebastian, S. Rani, S. Ray, C. Harrys Kishore, S. Kanth, M. Ahmed, M. Kashyap, R. Mohmood, Y. Ramachandra, V. Krishna, B. Rahiman, S. Mohan, P. Ranganathan, S. Ramabadran, R. Chaerkady, A. Pandey, Human Protein Reference Database--2009 update., Nucleic Acids Res. 37((Database issue)) (2009) D767-72.

[6] T.Y. Lee, H.D. Huang, J.H. Hung, H.Y. Huang, Y. Y.S., W. T.H., dbPTM: An information repository of protein post-translational modification, Nucleic Acids Research 34(D622-D627. ) (2006).

[7] T.U. Consortium, Reorganizing the protein space at the Universal Protein Resource (UniProt), Nucleic Acids Res. 40(D71-D75 ) (2012).

[8] M. Larkin, G. Blackshields, N. Brown, R. Chenna, P. McGettigan, H. McWilliam, F. Valentin, I. Wallace, A. Wilm, R. Lopez, J. Thompson, T. Gibson, D. Higgins, Clustal W and Clustal X version 2.0., Bioinformatics 23 (2007) 2947-2948. .

[9] A.M. Waterhouse, J.B. Procter, D.M.A. Martin, M. Clamp, G.J. Barton, Jalview Version 2 - a multiple sequence alignment editor and analysis workbench, Bioinformatics 25((9)) (2009) 1189-1191

[10] S.A. Beausoleil, J. Villen, S.A. Gerber, J. Rush, S.P. Gygi, A probability-based approach for high-throughput protein phosphorylation analysis and site localization, Nature biotechnology 24(10) (2006) 1285-92.

[11] A. Byron, J. Humphries, J. Askari, S. Craig, A. Mould, M. Humphries, Anti-integrin monoclonal antibodies, Journal of Cell Science 122 (2009) 4009-4011.

[12] Y. Mao, J. Schwarzbauer, Fibronectin fibrillogenesis, a cell-mediated matrix assembly process, Matrix Biol. 24( 6) (2005) 389-399.

[13] V. Vogel, Mechanotransduction involving multimodular proteins: converting force into biochemical signals., Annu Rev Biophys Biomol Struct. 35 (2006) 459-88.

[14] R.O. Hynes, The extracellular matrix: not just pretty fibrils, Science 326(5957) (2009) 1216-9.

[15] R. Pankov, K. Yamada, Fibronectin at a glance., J Cell Sci. 115((PT20)) (2002) 3861-3.
